# Prognostic impact of ^18^F-FDG PET/CT in pathologic stage II invasive ductal carcinoma of the breast: re-illuminating the value of PET/CT in intermediate-risk breast cancer

**DOI:** 10.1186/s40644-022-00519-6

**Published:** 2023-01-04

**Authors:** Hye Lim Park, Sea-Won Lee, Ji Hyung Hong, Jieun Lee, Ahwon Lee, Soo Jin Kwon, Sonya Youngju Park, Ie Ryung Yoo

**Affiliations:** 1 Division of Nuclear Medicine, Department of Radiology, Eunpyeong St. Mary’s Hospital, College of Medicine, The Catholic University of Korea, Seoul, South Korea; 2 Department of Radiation Oncology, Eunpyeong St. Mary’s Hospital, College of Medicine, The Catholic University of Korea, Seoul, South Korea; 3 Division of Medical Oncology, Department of Internal Medicine, Eunpyeong St. Mary’s Hospital, College of Medicine, The Catholic University of Korea, Seoul, South Korea; 4 Division of Medical Oncology, Department of Internal Medicine, Seoul St. Mary’s Hospital, College of Medicine, The Catholic University of Korea, Seoul, South Korea; 5 Department of Hospital Pathology, Seoul St. Mary’s Hospital, College of Medicine, The Catholic University of Korea, Seoul, South Korea; 6 Division of Nuclear Medicine, Department of Radiology, Seoul St. Mary’s Hospital, College of Medicine, The Catholic University of Korea, Seoul, South Korea

**Keywords:** Invasive ductal carcinoma, Breast cancer, PET/CT, Stage II, Prognosis

## Abstract

**Background:**

The aim of this study is to investigate the impact of ^18^F-FDG PET/CT on prognosis of stage II invasive ductal carcinoma (IDC) of the breast primarily treated with surgery.

**Methods:**

The clinical records of 297 consecutive IDC with preoperative PET/CT and pathologically staged II in surgery from 2013 to 2017 were retrospectively reviewed. The maximum standardized uptake value (SUVmax), peak standardized uptake value (SUVpeak), tumor-to-liver ratio (TLR), and metabolic tumor volume (MTV) were measured. Association of clinicopathologic factors (age, T stage, N stage, AJCC pathologic stage of IIA or IIB, pathologic prognostic stage, grade, hormonal receptor status, HER2 status, Ki-67, and adjuvant therapy) and PET parameters with DFS was assessed using the Cox proportional hazards model.

**Results:**

There were 35 recurrences and 10 deaths at a median follow-up of 49 months (range 0.8 ~ 87.3). All PET parameters were significantly associated with DFS in univariate analysis but in multivariate analysis, SUVpeak was the only factor significantly associated with DFS (hazard ratio 2.58, 95% confidence interval 1.29–5.15, *P* = 0.007). In cohorts with higher values of SUVpeak or TLR, patients who received adjuvant chemotherapy had significantly superior DFS.

**Conclusion:**

Metabolic parameters derived from preoperative PET/CT was significantly associated with recurrence in stage II IDC primarily treated with surgery. PET/CT can be a powerful prognostic tool in conjunction with novel staging systems and current biomarkers for patients undergoing contemporary therapy. Our results urge to reconsider the currently underestimated value of PET/CT confined to diagnostic aspect and to newly recognize its prognostic impact in these intermediate-risk breast cancer.

**Supplementary Information:**

The online version contains supplementary material available at 10.1186/s40644-022-00519-6.

## Background

Breast cancer accounts for 30% of female cancers diagnosed annually in the United States [1]. Although screening accelerated early diagnosis and significantly reduced mortality, breast cancer is still the leading cause of death due to malignancy among women worldwide and is designated a global health threat [2]. Imaging such as mammography, ultrasonography (US), and magnetic resonance imaging (MRI) plays an essential role in diagnosis of breast cancer although the final diagnosis is confirmed with biopsy [3].

According to the NCCN guideline Version 4.2022 [4], ^18^F-fluorodeoxyglucose (^18^F-FDG) positron emission tomography/computed tomography (PET/CT) for initial staging is not routinely recommended to clinical stage I, II, or operable III (T3N1) breast cancer, because of its low FDG-avidity in small or low-grade tumors, low sensitivity for axillary nodal staging, and low probability of distant metastasis. Although T3N0 is staged as IIB, patients with large breast tumors (> 5 cm) are preferred for pre-operative systemic therapy in order to facilitate breast conservation [3]. Curative surgery without pre-operative systemic therapy is recommended for stage I to II breast cancer with exception of T3N0 disease. The value of PET/CT remains undetermined for the intermediate-risk patients, especially stage II breast cancer with small tumors (< 5 cm) primarily treated with surgery.

With growing understanding of the biology of breast cancer, a novel prognostic staging system (pathologic prognostic staging [PPS]) has been developed in the most recent, 8th edition of AJCC (American Joint Committee on Cancer) by incorporating histologic grade, estrogen receptor (ER), and human epidermal growth factor receptor-2 (HER2) status to the traditional TNM staging system [5]. The superiority of the prognostic staging system has been validated in multiple large cancer registries in the United States [6, 7]. Although incorporating these biological factors into the traditional TNM staging system significantly enhanced the prognostic power, we recognized a scope for further improvement by additional integration of tumor metabolism to current system. Metabolic parameters from FDG PET/CT are prognosticators which highly reflect tumor biology. High FDG uptake has been proven to be associated with poor prognosis in cancers arising from numerous sites including the breast, lung, head & neck, colorectum, and lymphomas [8–12]. We hypothesized that metabolic parameters from the FDG PET/CT at initial staging may have additional prognostic impact in intermediate-risk invasive ductal carcinoma (IDC), especially in comparison with the novel pathologic prognostic staging.

We aimed to investigate the impact of PET/CT on prognosis of small (< 5 cm) pathologic stage II of the breast primarily treated with surgery. To the best of our knowledge, this is the first study to demonstrate association between metabolic parameters derived from baseline PET/CT in these pathologically staged, intermediate-risk cohort of pure IDC histology. We also analyzed the effect of additional adjuvant treatment on the outcome of patients with higher values of PET parameters.

## Methods

### Patients

The clinical records of all consecutive female breast cancer who underwent primary surgery from 2013 to 2017 in single institution were retrospectively reviewed. Inclusion criteria were as follows: 1) histologically confirmed invasive ductal carcinoma (IDC), 2) acquisition of ^18^F-FDG PET/CT at diagnosis, 3) primarily treated with complete surgical resection, and 4) pathologic stage II on surgical specimen according to the American Joint Committee on Cancer (AJCC) staging system, 8th edition. Exclusion criteria were: 1) ductal carcinoma in situ, 2) histological type other than pure IDC, such as mixed type of IDC, 3) primary tumor > 5 cm (T3), 4) bilateral breast cancer, 5) presence of second malignancy, 6) delivery of neoadjuvant chemotherapy, and 7) tumors unfeasible for contouring of region of interest (ROI) due to several regions including low activities indistinguishable from surrounding parenchyma (Supplement 1). All procedures of this study were approved by the Institutional Review Board (IRB No. KC20RISI0816) with waiver of informed consent.

### ^18^F-FDG PET/CT protocol and imaging analysis

All patients fasted for at least 6 hours before the ^18^F-FDG PET/CT scan. A dose of 3.7–5.5 MBq/kg ^18^F-FDG was injected intravenously, and scanning began 60 minutes later. None of the patients had a blood glucose level > 150 mg/dL. No intravenous contrast agent was administered. Images were acquired using a combined PET/CT in-line system (Biograph Duo, Biograph TruePoint, Siemens Medical Solutions, Knoxville, TN, USA; and Discovery 710D, GE Healthcare, Milwaukee, WI, USA). The acquisition time was 2–3 minutes per bed position. All patients were in supine position during PET/CT scanning. Non-contrast-enhanced CT began at the orbitomeatal line and progressed to the proximal thigh using a standard protocol: 130 kVp, 80 mAs, 5-mm slice thickness (Biograph Duo); and 120 kVp, 50 mAs, 5-mm slice thickness (Biograph TruePoint); and 120 kVp, variable mAs adjusted by topographic image, 2.5-mm slice thickness (Discovery 710D). PET scans of the same body region followed immediately. The CT data were used for attenuation correction, and PET images were reconstructed using a standard ordered-subset expectation maximization algorithm with 4 iterations and 8 subsets.

PET/CT data were interpreted by two board-certified nuclear medicine specialists (HLP and SYP) with over 10 years of experience. All PET/CT scans were assessed using a single software (XD3, Mirada Medical, Oxford, UK). For the semi-quantitative analysis, the maximum standardized uptake value (SUVmax) and peak standardized uptake value (SUVpeak) of PET were measured by visually placing the region of interest around the site of increased FDG uptake of primary cancer. For multiple breast IDC, FDG uptake of the largest tumor was measured. In order to compensate for the difference arising from three different scanners, correction by mean SUV of liver (3 cm^3^-sized volume of interest on right hepatic lobe) was used to compute tumor-to-liver ratio (TLR). The TLR was calculated as the ratio of tumor SUVmax to mean SUV of the patient’s liver. We also measured metabolic tumor volume (MTV) of primary tumor using a cut-off of SUV 2.5.

### Analysis of clinicopathologic factors

The factors known to be associated with prognosis of breast cancer including T stage, N stage, histologic grade, expression of ER, progesterone receptor (PR), HER2, and Ki-67 were analyzed by board-certified pathologists. The Nottingham modification of the Scarff-Bloom-Richardson grading system was used for histologic grading [13]. ER and PR testing was performed in line with standard procedures [14]. HER-2 positivity was defined using the following criteria: 1) overexpression of C-erbB2 with an immunohistochemistry (IHC) of 3+; 2) HER2 gene amplification on fluorescence in situ hybridization (FISH) or silver-enhanced in situ hybridization (SISH) in case of C-erbB2 IHC score of 2+ [15]. The molecular subtypes were determined according to the St. Gallen classification [16]. Pathologic prognostic stage (PPS) was determined as described in the 8th edition of AJCC Staging Manual and categorized into stages I, II, and III [5]. The Ki-67 labeling index was classified as low or high with the cut-off of 14% [17].

### Treatment and follow-up

Primary surgery was determined with surgeon’s discretion and both breast conserving surgery as well as mastectomy were allowed. Axillary staging was performed with either sentinel lymph node biopsy or axillary dissection. Hormonal therapy, HER2-targeted therapy, and chemotherapy were delivered according to the contemporary guideline based on molecular status in addition to patient- and tumor-related factors. Postoperative radiation therapy (RT) was delivered to whole breast or chest wall and/or regional nodes including axillary, infraclavicular, supraclavicular, and/or internal mammary area according to the guidelines [4]. RT was sequenced after adjuvant chemotherapy.

Follow-up was scheduled at every 3 months for the first year after surgery. Then the patients visited with 6-month interval until 2–3 years after surgery and then yearly thereafter. Clinical interview, physical examination, serum tests, and imaging with mammography, ultrasonography, computed tomography, magnetic resonance imaging, and bone scan or PET/CT were performed according to schedule. Cases suspicious of recurrence in imaging were histologically confirmed with biopsy if accessible.

### Statistical analyses

All continuous values of PET parameters, or metabolic parameters, including SUVmax, SUVpeak, TLR, and MTV are described as mean ± standard deviation (SD) (range). Student’s *t*-test and Mann-Whitney test were performed to compare the values of PET parameters according to different variables. Disease-free survival (DFS) was defined as the time from date of primary surgery until the first evidence of recurrence or death. Overall survival (OS) was defined as the time between primary surgery and death from any cause. The maximally selected chi-square test (‘maxstat’ package) was used to determine the cut-off levels for PET parameters which optimally discriminated DFS (R software, version 4.0.4, R for Statistics Computing, Vienna, Austria). It is a method which divides the sample into two groups by calculating the cut-off value with statistically most significant difference in survival outcome [18]. DFS was estimated using the Kaplan-Meier method with log-rank test. Univariate and multivariate analyses of association between prognostic factors and DFS was performed with Cox proportional hazards regression with stepwise forward selection of variables. Factors with *P* <  0.05 in univariate analysis were included in multivariate analysis. To assess the efficacy of adjuvant chemotherapy, subgroup analysis of patients with higher values of metabolic parameters above the cut-off levels were conducted by comparing their DFS according to the administration of adjuvant chemotherapy. *P*-values < 0.05 were considered to indicate statistical significance. Statistical analyses were performed using IBM SPSS (Statistical Package for Social Sciences) software for Windows, version 24.0 (IBM Corp., Armonk, NY, USA).

## Results

### Demographics and metabolic parameters at baseline

There were 297 pathologic stage II IDC patients with preoperative PET/CT who underwent complete resection (Table 1). Median tumor size was 2.4 cm (range: 0.9–5). The mean values of SUVmax, SUVpeak, TLR, and MTV were significantly higher in subgroups with poor prognostic factors for T stage, histologic grade, ER, PR, HER2, and Ki-67 (Table 1). The number of patients included in this study for each subtype of breast cancer were 174 luminal A, 25 luminal B, 30 HER2-enriched, and 68 triple negative, respectively. Hormonal therapy, HER2-targeted therapy, and adjuvant chemotherapy were delivered to 181, 49, and 274 patients, respectively. The mean values of SUVmax, SUVpeak, and TLR did not differ between stage IIA and stage IIB of the conventional TNM staging system while mean MTV was significantly higher in stage IIB compared to stage IIA. The mean values of SUVmax, SUVpeak, TLR, and MTV significantly increased with higher pathologic prognostic stage. The mean values of SUVmax, SUVpeak, TLR, and MTV were higher in patients treated with HER2-targeted therapy and were lower in those who received hormonal therapy.

**Table 1:** Demographics and PET parameters at baseline

	Total N (%)	SUVmax Mean ± SD	***P***	SUVpeak Mean ± SD	***P***	TLR Mean ± SD	***P***	MTV (cm^**3**^) Mean± SD	***P***
	297 (100)	7.57 ± 4.41		5.49 ± 3.42		4.72 ± 5.50		3.42 ± 2.00	
**Age, years**			0.379		0.380		0.174		0.784
< 40	35 (11.8)	8.08 ± 4.35		5.85 ± 3.29		3.79 ± 1.97		4.04 ± 3.38	
≥ 40	262 (88.2)	7.50 ± 4.42		5.44 ± 3.44		3.37 ± 2.01		4.81 ± 5.73	
**T stage**			< 0.001		< 0.001		< 0.001		< 0.001
T1	59 (19.9)	5.87 ± 3.25		3.72 ± 2.18		2.63 ± 1.41		1.40 ± 1.54	
T2	238 (80.1)	7.99 ± 4.56		5.93 ± 3.53		3.62 ± 2.08		5.55 ± 5.82	
**N stage**			0.112		0.018		0.089		< 0.001
N0	150 (50.5)	7.99 ± 4.69		5.95 ± 3.63		3.62 ± 2.13		5.59 ± 5.74	
N1	147 (49.5)	7.14 ± 4.07		5.02 ± 3.14		3.22 ± 1.85		3.84 ± 5.12	
**TNM stage**			0.225		0.081		0.225		0.034
IIA	209 (70.4)	7.39 ± 4.43		5.32 ± 3.43		3.34 ± 2.00		4.41 ± 5.28	
IIB	88 (29.6)	7.99 ± 4.35		5.89 ± 3.38		3.62 ± 2.01		5.47 ± 5.97	
**Pathologic prognostic stage**			< 0.001		< 0.001		< 0.001		0.005
I	167 (38.4)	6.29 ± 3.34		4.52 ± 2.46		2.87 ± 1.53		3.70 ± 4.72	
II	113 (17.8)	9.10 ± 4.98		6.55 ± 3.91		4.09 ± 2.29		5.69 ± 5.97	
III	17 (32.3)	9.98 ± 5.46		7.90 ± 4.77		4.47 ± 2.36		8.29 ± 6.98	
**Grade**			< 0.001		< 0.001		< 0.001		< 0.001
1	43 (14.5)	5.16 ± 2.90		3.67 ± 2.16		2.36 ± 1.33		2.33 ± 3.09	
2	110 (37)	6.21 ± 3.24		4.44 ± 2.43		2.79 ± 1.46		3.76 ± 4.94	
3	144 (48.5)	9.33 ± 4.84		6.83 ± 3.83		4.22 ± 2.21		6.17 ± 6.06	
**ER**			< 0.001		0.001		< 0.001		< 0.001
Positive	194 (65.3)	6.32 ± 3.36		4.55 ± 2.52		2.86 ± 1.51		3.98 ± 5.16	
Negative	103 (34.7)	9.92 ± 5.14		7.25 ± 4.14		4.48 ± 2.36		6.12 ± 5.88	
**PR**			< 0.001		< 0.001		< 0.001		< 0.001
Positive	178 (59.9)	6.39 ± 3.34		4.58 ± 2.47		2.91 ± 1.52		3.70 ± 4.65	
Negative	119 (40.1)	9.34 ± 5.17		6.85 ± 4.13		4.20 ± 2.36		6.25 ± 6.30	
**HER2**			< 0.001		< 0.001		< 0.001		0.016
Positive	55 (18.5)	9.12 ± 4.20		6.49 ± 2.96		4.29 ± 2.14		5.42 ± 5.12	
Negative	242 (81.5)	7.22 ± 4.39		5.26 ± 3.48		3.23 ± 1.92		4.56 ± 5.59	
**Luminal type**			0.139		0.115		0.162		0.103
Luminal A	174 (58.6)	7.44 ± 4.68		5.46 ± 3.59		3.38 ± 2.14		4.79 ± 5.75	
Luminal B	25 (8.4)	6.82 ± 3.79		4.63 ± 2.52		3.07 ± 1.63		2.69 ± 2.24	
HER2 positive	30 (10.1)	6.71 ± 2.73		4.62 ± 2.23		2.99 ± 1.11		3.50 ± 4.18	
Triple negative	68 (22.9)	8.56 ± 4.39		6.25 ± 3.57		3.85 ± 2.03		5.83 ± 5.96	
**Ki-67**			< 0.001		< 0.001		< 0.001		0.031
< 14%	35 (11.8)	5.19 ± 2.65		3.70 ± 1.98		2.30 ± 1.14		3.99 ± 6.54	
≥ 14%	262 (88.2)	7.89 ± 4.50		5.73 ± 3.50		3.57 ± 2.05		4.82 ± 5.36	
**Hormonal therapy**			< 0.001		< 0.001		< 0.001		< 0.001
Yes	181 (60.9)	6.34 ± 3.27		4.53 ± 2.46		2.86 ± 1.45		3.71 ± 4.55	
No	116 (39.1)	9.50 ± 5.21		6.99 ± 4.11		4.30 ± 2.40		6.30 ± 6.43	
**HER2-targeted therapy**			0.001		0.003		< 0.001		0.027
Yes	49 (16.5)	9.17 ± 4.22		6.44 ± 2.95		4.28 ± 2.14		5.36 ± 5.12	
No	248 (83.5)	7.26 ± 4.38		5.30 ± 3.48		3.25 ± 1.93		4.60 ± 5.58	
**Adjuvant chemotherapy**			0.128		0.051		0.178		0.012
Yes	274 (92.3)	7.46 ± 4.37		5.37 ± 3.36		3.38 ± 1.99		4.46 ± 5.18	
No	23 (7.7)	8.91 ± 4.74		6.85 ± 3.84		3.92 ± 2.08		7.90 ± 7.98	

### Metabolic parameters and recurrence

There were total 35 patients (11.8%) with recurrence at the time of analysis (Supplement 2). The values of SUVmax (9.41 ± 5.02 vs. 7.33 ± 4.27; *P* = 0.012), SUVpeak (7.16 ± 4.18 vs. 5.26 ± 3.25; *P* = 0.004), TLR (4.31 ± 2.35 vs. 3.31 ± 1.93; *P* = 0.027), and MTV (7.28 ± 7.24 vs. 4.38 ± 5.15; *P* = 0.002) of recurrent patients were significantly higher than the corresponding values of patients free of disease (Table 2). The SUVmax (11.18 ± 6.34; *P* = 0.021), SUVpeak (8.30 ± 5.57; *P* = 0.035), and TLR (5.00 ± 2.85; *P* = 0.026) of the patients with locoregional recurrence were significantly higher than those of patients without recurrence (Table 2). The SUVpeak (6.49 ± 3.05; *P* = 0.033) and MTV (7.96 ± 8.55; *P* = 0.011) of 22 patients with distant recurrence including 1 patient with concomitant regional recurrence at axillary LN were also significantly higher than those of patients without recurrence.

**Table 2:** Comparison of PET parameters according to presence of recurrence

Parameters	No evidence of recurrence (***N*** = 262)	Recurrence (N = 35)	***P***	Locoregional recurrence (***N*** = 13)	***P***	Distant metastasis (***N*** = 22)	***P***
SUVmax	7.33 ± 4.27	9.41 ± 5.02	0.012	11.18 ± 6.34	0.021	8.35 ± 3.84	0.133
SUVpeak	5.26 ± 3.25	7.16 ± 4.18	0.004	8.30 ± 5.57	0.035	6.49 ± 3.05	0.033
TLR	3.31 ± 1.93	4.31 ± 2.35	0.027	5.00 ± 2.85	0.026	3.90 ± 1.95	0.108
MTV (cm^3^)	4.38 ± 5.15	7.28 ± 7.24	0.002	6.10 ± 4.94	0.071	7.96 ± 8.55	0.011

### Metabolic parameters and prognosis

**Fig. 1:**
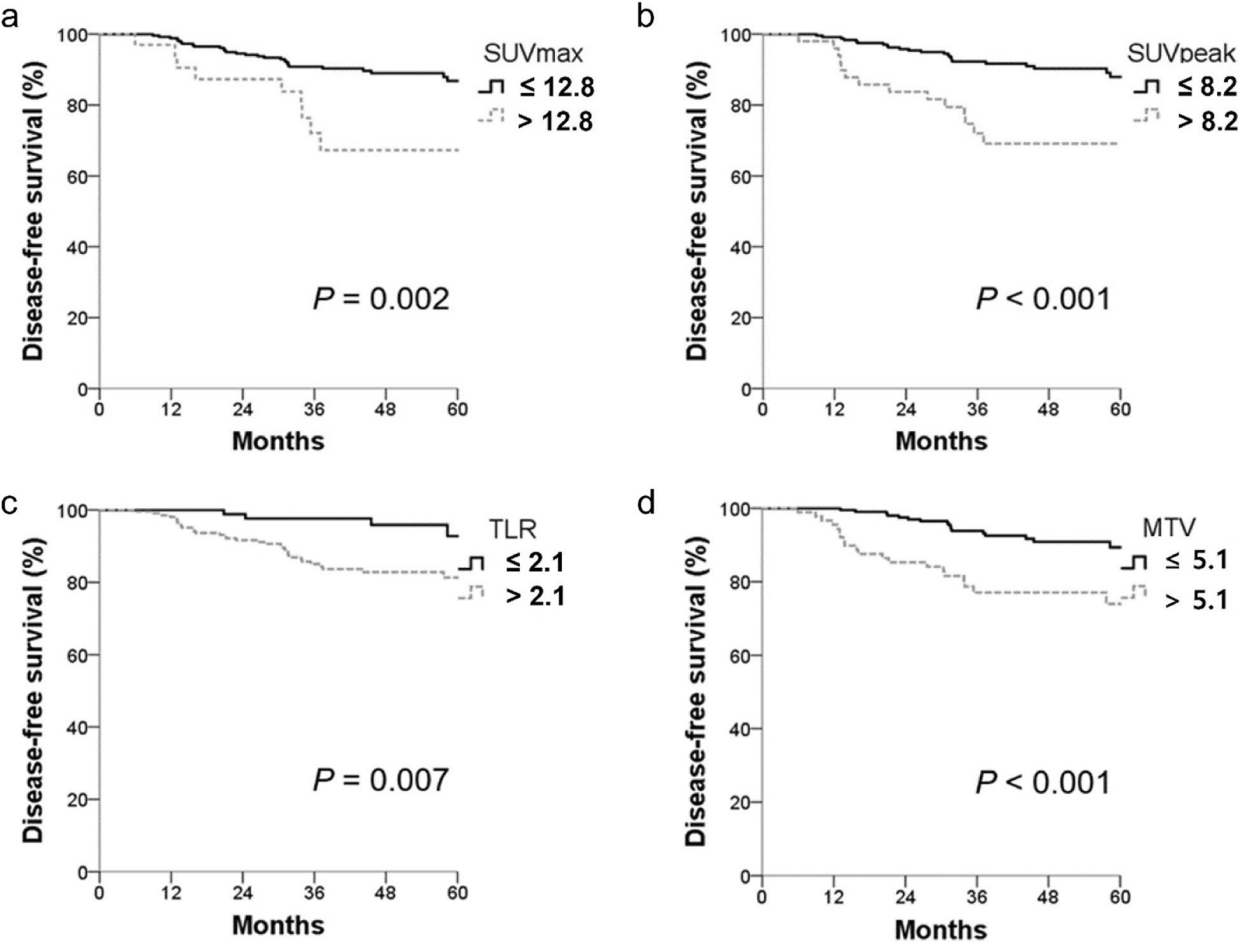
Disease-free survival according to metabolic parameters

Median follow-up was 49 months (range, 0.8–87.3). There were total 10 deaths at the time of analysis. The 4-year OS rate was 96.2% for all patients. Median OS was not reached, and mean OS was 84.4 months (95% confidence interval [CI], 82.7–86). Likewise, median DFS was also not reached and mean DFS was 78.2 months (95% CI, 75.6–80.9). The 4-year DFS rate was 86.6% for all patients. All of the metabolic parameters derived from initial PET/CT including SUVmax, SUVpeak, TLR, and MTV significantly discriminated DFS with the cut-off levels obtained using the maximal chi-square method, of which are shown in Fig. 1. The 4-year DFS rate for SUVmax > 12.8 was lower compared that of SUVmax ≤12.8, which were 67.3 and 89%, respectively (*P* = 0.002). The 4-year DFS rate was 69.1% for patients with SUVpeak > 8.2, significantly poor compared to 90.3% of those with SUVpeak ≤8.2 (*P* <  0.001). Similarly, the 4-year DFS rates were 82.8% for TLR > 2.1 and 95.8% for TLR ≤ 2.1, respectively (*P* = 0.007). The 4-year DFS rate for MTV > 5.1 was lower compared that of MTV ≤ 5.1, which were 77 and 90.8%, respectively (*P* <  0.001).

### Factors associated with DFS

In univariate analysis, all of the metabolic parameters were significantly associated with DFS (Table 3). Patients with higher values of metabolic parameters above the cut-off levels were over 3 times more likely to experience recurrence. Higher pathologic prognostic stage was associated with greater risk of recurrence. Those who underwent hormonal therapy and adjuvant chemotherapy had lower probability of recurrence.

**Table 3:** Factors associated with disease-free survival on the Cox proportional hazards model

	Disease-free survival			
	Univariate		Multivariate	
	Hazard ratio (95% CI)	***P***	Hazard ratio (95% CI)	***P***
**Age, years**		0.451		
≥ 40	1.00 (reference)			
< 40	1.58 (0.48–5.13)			
**Ki-67**		0.426		
< 14	1.00 (reference)			
≥ 14	1.62 (0.50–5.26)			
**Hormonal therapy**		0.011		0.639
Yes	1.00 (reference)		1.00 (reference)	
No	2.31 (1.21–4.44)		1.31 (0.43–3.97)	
**HER2-targeted agent**		0.570		
Yes	1.00 (reference)			
No	1.75 (0.62–4.95)			
**Adjuvant chemotherapy**		0.014		0.051
Yes	1.00 (reference)		1.00 (reference)	
No	3.28 (1.27–8.45)		2.58 (0.99–6.68)	
**TNM stage**		0.109		
IIA	1.00 (reference)			
IIB	1.71 (0.89–3.30)			
**Pathologic prognostic stage**		0.046		0.324
I	1.00 (reference)		1.00 (reference)	
II	1.68 (0.84–3.37)		0.99 (0.33–2.98)	
III	3.82 (1.39–10.51)		2.19 (0.50–9.66)	
**SUVmax**		0.004		0.948
≤ 12.8	1.00 (reference)		1.00 (reference)	
> 12.8	3.03 (1.43–6.43)		1.04 (0.34–3.16)	
**SUVpeak**		< 0.001		0.007
≤ 8.2	1.00 (reference)		1.00 (reference)	
> 8.2	3.46 (1.78–6.74)		2.58 (1.29–5.15)	
**TLR**		0.012		0.078
≤ 2.1	1.00 (reference)		1.00 (reference)	
> 2.1	3.81 (1.35–10.77)		2.65 (0.90–7.82)	
**MTV (cm**^**3**^**)**		0.001		0.241
≤ 5.1	1.00 (reference)		1.00 (reference)	
> 5.1	3.10 (1.63–5.93)		1.60 (0.73–3.49)	

**Fig. 2:**
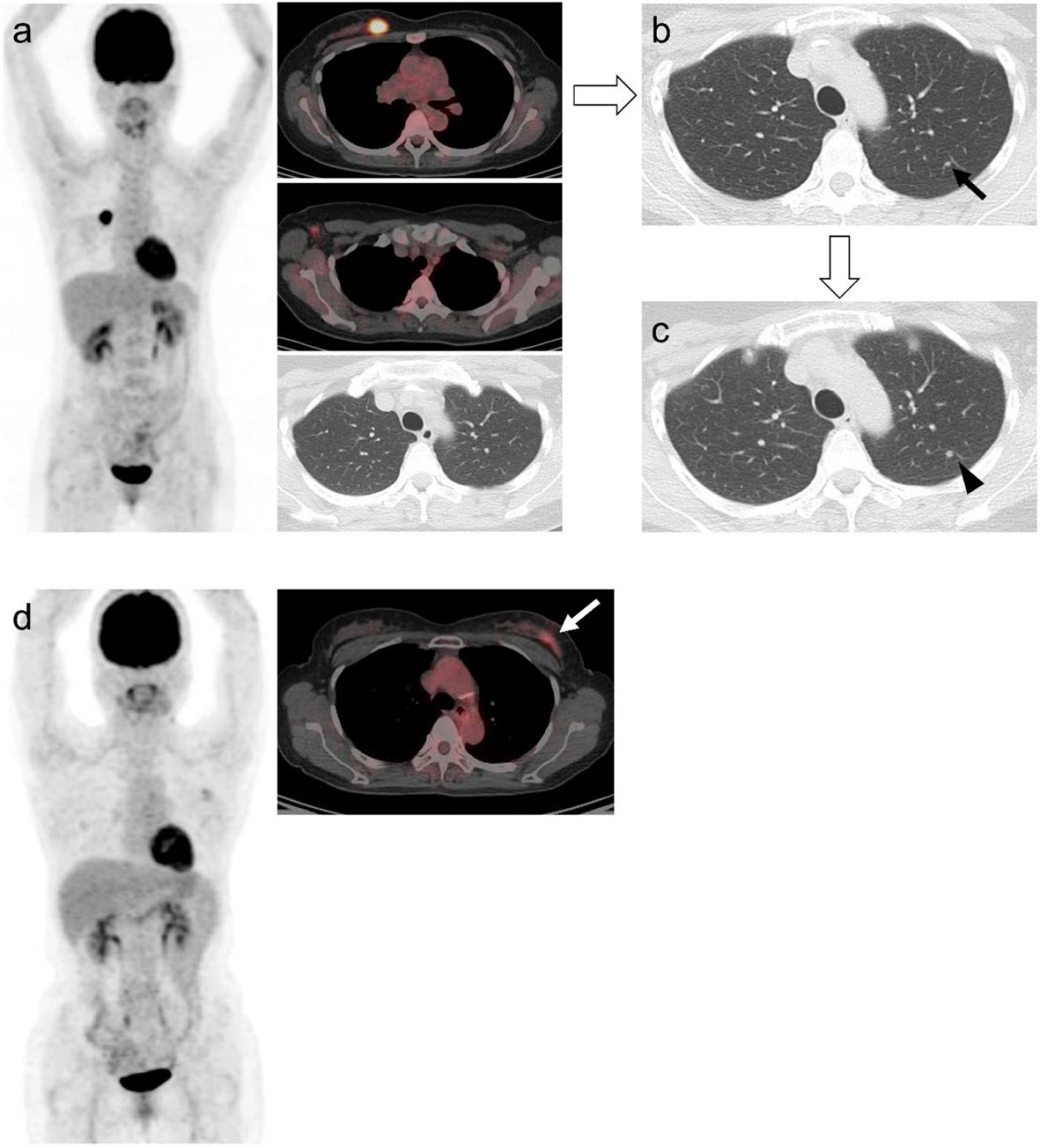
Case comparison according to PET parameters between two breast cancer patients with identical pathologic prognostic stage (IB). **a**^18^F-FDG PET/CT scan of a 46 year-old female revealed right breast malignancy with intense FDG uptake (SUVmax 13.6, SUVpeak 10.9, MTV 5.9 cm^3^ and TLR 7.2). Right axillary lymph node was pathologically negative for malignancy. **b** Sixteen months after modified radical mastectomy, a small nodule was seen in left upper lung (**c**) which grew in size in 2 months, suggestive of metastasis. **d** A 66 year-old patient underwent PET/CT for breast cancer staging and it showed mild FDG uptake in left breast with SUVmax 3.2, SUVpeak 1.9, MTV 0.1 cm^3^, and TLR 1.5. She had no evidence of recurrence during 37 months of follow-up

In multivariate analysis, SUVpeak was the only factor independently associated with DFS (Table 3 and Fig. 2). Patients with SUVpeak > 8.2 were more than twice as likely (hazard ratio [HR] 2.58, 95% CI 1.29–5.15) to experience relapse compared to those with SUVpeak ≤8.2 (*P* = 0.007). Likewise, although the patients with TLR > 2.1 had a higher hazard ratio (HR 2.65) than TLR ≤ 2.1, the difference was marginal (*P* = 0.078). MTV > 5.1 had only numerically greater risk of recurrence compared to MTV ≤ 5.1. Patients who did not receive adjuvant chemotherapy were more than twice as more likely to recur (HR 2.58, 95% CI 0.99–6.68) than those who did receive chemotherapy, with marginal significance (*P* = 0.051).

### Effect of adjuvant chemotherapy on patients with higher values of metabolic parameters

**Fig. 3:**
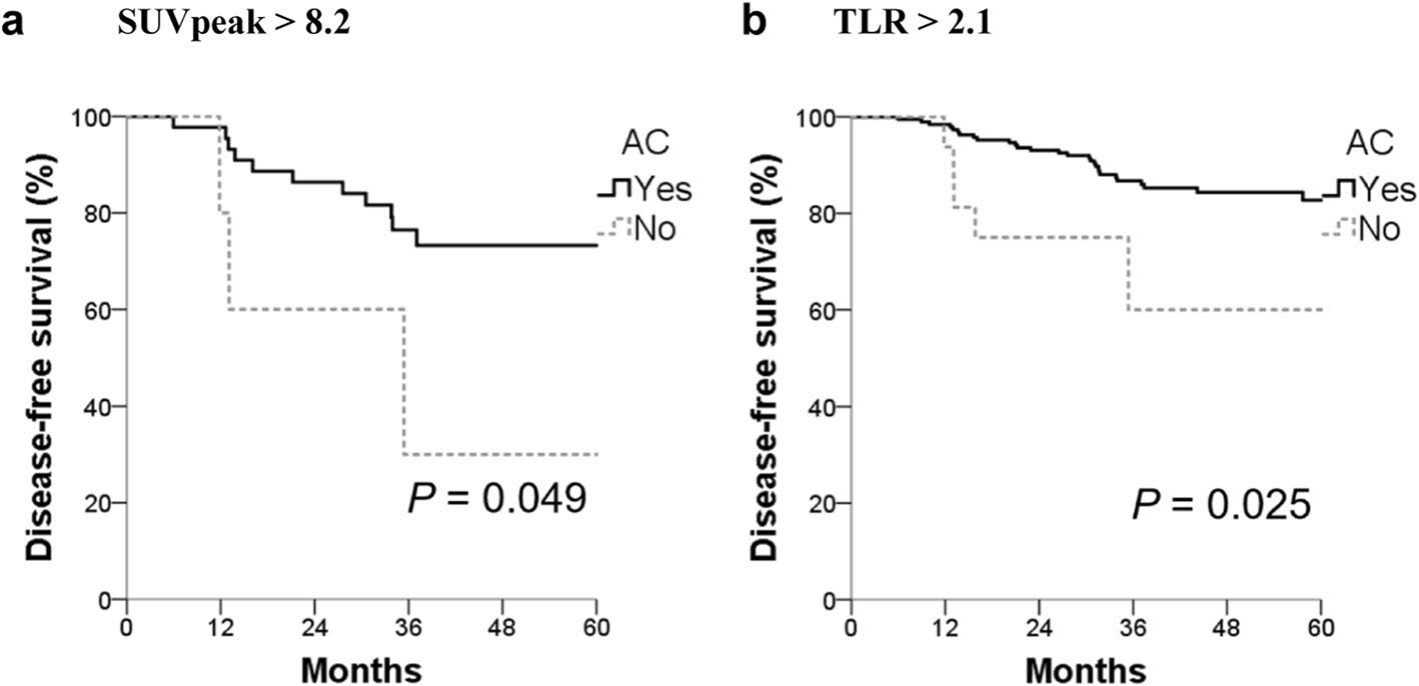
Disease-free survival in patients with higher SUVpeak subgroup and higher TLR subgroup

Adjuvant chemotherapy was delivered to 274 (92.3%) patients. Among them, 163 (59.5%) were luminal A, 25 (9.1%) were luminal B, 30 (10.9%) were HER2 positive, and 56 (20.4%) were triple negative. The DFS of the subset of patients with values of metabolic parameters above the cut-off levels were separately analyzed (SUVmax > 12.8 [*N* = 34], SUVpeak > 8.2 [*N* = 51], TLR > 2.1 [*N* = 211], and MTV > 5.1 [*N* = 92]). Patients who received adjuvant chemotherapy had superior DFS compared to those who did not in patients with higher values of metabolic parameters (Fig. 3). For patients with SUVpeak > 8.2, the 4-year DFS rate was 73.3% for those who underwent chemotherapy, significantly better compared to 30% of those who did not (*P* = 0.049). For patients with TLR > 2.1, the 4-year DFS rate for patients who received chemotherapy was 84.3%, significantly higher than 60% of those who did not (*P* = 0.025). For patients with MTV > 5.1 (*P* = 0.141) and SUVmax > 12.8 (*P* = 0.262), patients who underwent chemotherapy had better DFS than those did not, but the difference was only numerical.

## Discussion

^18^F-FDG PET/CT is seldom recommended to clinically non-metastatic breast cancer as a routine for primary workup [3]. Although addition of PET/CT at primary workup may upstage initial stage and influence ensuing treatment strategy especially in locally advanced breast cancer, the clinical role of ^18^F-FDG PET/CT in intermediate-risk breast cancer has not been established to date [19, 20]. In this study, we evaluated the role of PET/CT in pathologic stage II breast cancer undergoing primary resection and obtained notable results. Higher SUVmax, SUVpeak, TLR, and MTV levels at baseline PET/CT were significantly associated with poor clinicopathologic prognostic factors such as large tumor size, higher pathologic prognostic stage, higher tumor grade, negative hormonal receptor, positive HER2 status, and higher Ki-67, all of which are in line with previous reports [21]. The values of PET parameters were also significantly higher in tumors with recurrence compared to those without recurrence. Although higher SUVmax, SUVpeak, TLR, or MTV were associated with poorer DFS in univariate analysis, SUVpeak was the only prognostic factor significantly related to DFS even after adjustment for other prognostic factors in multivariate analysis. These findings suggest that metabolic parameters obtained from baseline PET/CT well demonstrate the tumor biology and consequent outcome of breast cancer. Such results are consistent with previous studies reporting the prognostic value of PET/CT in predicting tumor recurrence. In those studies, SUVmax was the most commonly used parameter, but significance of other parameters such as SUVpeak, TLG, and MTV also have been reported [8, 22–24]. SUVpeak is defined as an average SUV over 1 cm^3^ of volume of interest with greatest activity. However, it may not necessarily contain the hottest pixel value. It is less affected by noise and scan time for small tumors in particular, thus often considered as the most reliable parameter for FDG PET quantification [25, 26]. Its reliability as a parameter for quantification demonstrated in this study as significant correlation with multiple clinicopathologic risk factors as well as DFS may be attributable at least in part to the relatively small tumor volume of this study.

One of the limitations of previous studies is that mixed cohort of patients with stage III or large tumors were also included. Large tumors sized > 5 cm (T3) are indicated for neoadjuvant chemotherapy in breast conserving therapy, so we excluded T3 tumors. Against this background, we focused on the more specific cohort of surgically staged stage II invasive ductal carcinoma with small (≤ 5 cm) tumors and included T1–2 N0–1 disease only. After primary resection and adjuvant radiotherapy, relatively small stage II breast cancer patients who do not receive neoadjuvant chemotherapy are considered for hormonal therapy, HER2-targeted therapy, or systemic chemotherapy according to surgical pathology and molecular subtype. In contrast to the evident indications for hormonal therapy or HER2-targeted therapy, who will most benefit from adjuvant chemotherapy is still under active investigation.

The oncologic outcome of this study (4-year OS of 96% and 4-year DFS of 87%) was comparable to those of previous studies. The ACOSOG Z0011 trial (5-year OS of 92% and 5-year DFS of 83%) has inclusion criteria analogous to ours of small breast cancer except for excluding N0 and including N1 only [27]. According to the most recent version of the AJCC staging manual, the 5-year disease-specific survival of T1–2 N0–1 patients range from 85 to 98% [28]. When these stage II patients with small tumors are followed long enough, a non-negligible proportion of them may experience recurrence eventually [29], therefore requiring criteria for selection of patients who would most benefit from adjuvant chemotherapy from early in the course of treatment.

The value of PET/CT in these T1–2 N0–1 patients have been underrated due to their low risk of extra-axillary disease. Instead of confining the value of PET/CT to diagnostic role, we demonstrated the prognostic significance of PET/CT in these intermediate-risk patients by showing strong association between metabolic parameters and clinical outcome. Moreover, adjuvant chemotherapy significantly improved DFS in subgroup of patients with higher SUVpeak or TLR in our data, which suggests that PET parameters can provide clinicians with a chance to consider adjuvant treatment according to the tumor metabolism assessed with baseline PET/CT before initiation of treatment.

The outcome of breast cancer has been conventionally prognosticated using the TNM staging system. Despite its clinical convenience, there were disparities in outcome of patients within the same stage. This limitation has driven extensive research worldwide on the biology of breast cancer including hormonal receptor and HER2 to begin with, and are still actively ongoing. Most recently, a novel staging system with histologic grade, estrogen receptor, and HER2 status in addition to the traditional TNM staging has been included in the newest version of AJCC Staging Manual as the prognostic staging system [5]. Stage II by TNM stage is further classified into stages IB to IIIB by prognostic staging comprised of the above molecular markers. The final markers included in the new prognostic staging system were selected through numerous processes of verification. The leading groups that formulated the prognostic staging system validated its superiority using the data of large cohorts treated with primary surgery and modern adjuvant therapy from the SEER data, the California Cancer Registry, and the National Cancer Data Base [6, 7]. Although prognostic staging system is currently the most comprehensively devised and most thoroughly validated tool for prognostication, the molecular markers adopted in this staging system remain different facets of the biology of breast cancer. Addition of the metabolic parameters measured from baseline PET/CT can offer a collective understanding of tumor biology through integration of tumor metabolism into the biologic markers included in the prognostic staging system. According to our data, preoperative PET parameters better correlated with any of the prognostic factors identified in the final surgical pathology including the pathologic prognostic stage. In univariate analysis, in addition to PET parameters, pathologic prognostic stage was significantly related to DFS while TNM stage failed to show significant relation with DFS. In multivariate analysis, SUVpeak was the only parameter independently associated with DFS, showing stronger association with DFS than pathologic prognostic stage or TNM stage.

The mechanism in which PET parameters reflect tumor outcome may be explained with the elevated level of metabolic activity of cancer cells, thus requiring more glucose than normal cells. In order to facilitate glucose uptake, cancer cells express higher levels of glucose transporter (GLUT), especially GLUT1 [30]. GLUT1 transports ^18^F-FDG, a glucose analog, into cancer cells which helps visualize tumor in PET/CT. In breast cancer, GLUT1 is reported to be expressed more in higher grade and rapidly proliferating tumor cells, such as basal-like cells [31, 32]. GLUT1 expression has been related to invasiveness and poor differentiation of breast cancer [33]. There also have been reports of higher GLUT1 expression in poor prognostic subsets such as triple-negative breast cancer [34]. The overexpression of GLUT1 has shown association with tumorigenesis and hypoxic tumor microenvironment [35, 36]. A recent bioinformatics study which analyzed major datasets including The Cancer Genome Atlas (TCGA), Gene Expression Omnibus (GEO), and Gene Expression Profiling Interactive Analysis (GEPIA) demonstrated poor relapse-free survival and overall survival in breast cancer with higher level of GLUT1 expression [31]. These findings from previous studies at molecular and genetic levels were metabolically corroborated in this study.

The limitation of this study owes to the retrospective design within single center experience. Potential biases include uneven selection of patients and slight differences in semi-quantitative measurements due to the analysis of more than one PET/CT systems. In an effort to standardize the latter, TLR was used for adjustment. The tumor biology of IDCs with very low FDG uptake could not be sufficiently incorporated because cases unfeasible for definite delineation of ROI in PET/CT were excluded. Considering the indolent nature of early breast cancer, our follow-up of median 4 years requires further maturation of data. However, we deemed the follow-up period of this study adequate for analysis of DFS since majority of recurrences occur within the first few years. Because this study was designed before the routine use of relatively recent gene test panels such as Oncotype Dx or Mammaprint, they were not included in this study regarding high level validation of these assays is still accumulating. For the same reason, they were not mandatory for the prognostic staging system [5]. However, there has been a report of higher levels of SUVmax related to higher Oncotype Dx recurrence scores [37], which suggests a direction for future studies. Despite the above limitations, this study has successfully demonstrated the prognostic value of initial PET/CT in a pure cohort of pathologic stage II IDC by showing significant association between metabolic parameters and recurrence.

## Conclusions

Metabolic parameters derived from baseline PET/CT was significantly associated with recurrence in stage II IDC of the breast primarily treated with surgery. PET/CT can be a powerful tool for prognostication in conjunction with novel staging systems and current biomarkers for patients undergoing contemporary therapy. Our results urge to reconsider the currently underestimated value of PET/CT confined to diagnostic aspect and to newly recognize its prognostic impact in these intermediate-risk breast cancer.

## Supplementary Information

**Additional file 1:** **Supplement 1.** Flowchart of study population.

**Additional file 2: Supplement 2.** Characteristics of recurrence (*N* = 35).

## Data Availability

The datasets analyzed during the current study are available from the corresponding author on reasonable request.
