# Supplementary figures and images for: Prognostic impact of 18F-FDG PET/CT in pathologic stage II invasive ductal carcinoma of the breast: re-illuminating the value of PET/CT in intermediate-risk breast cancer
Source: Cancer Imaging. 2023 Jan 4;23:2. doi: 10.1186/s40644-022-00519-6 (PMC9811771; doi:10.1186/s40644-022-00519-6)

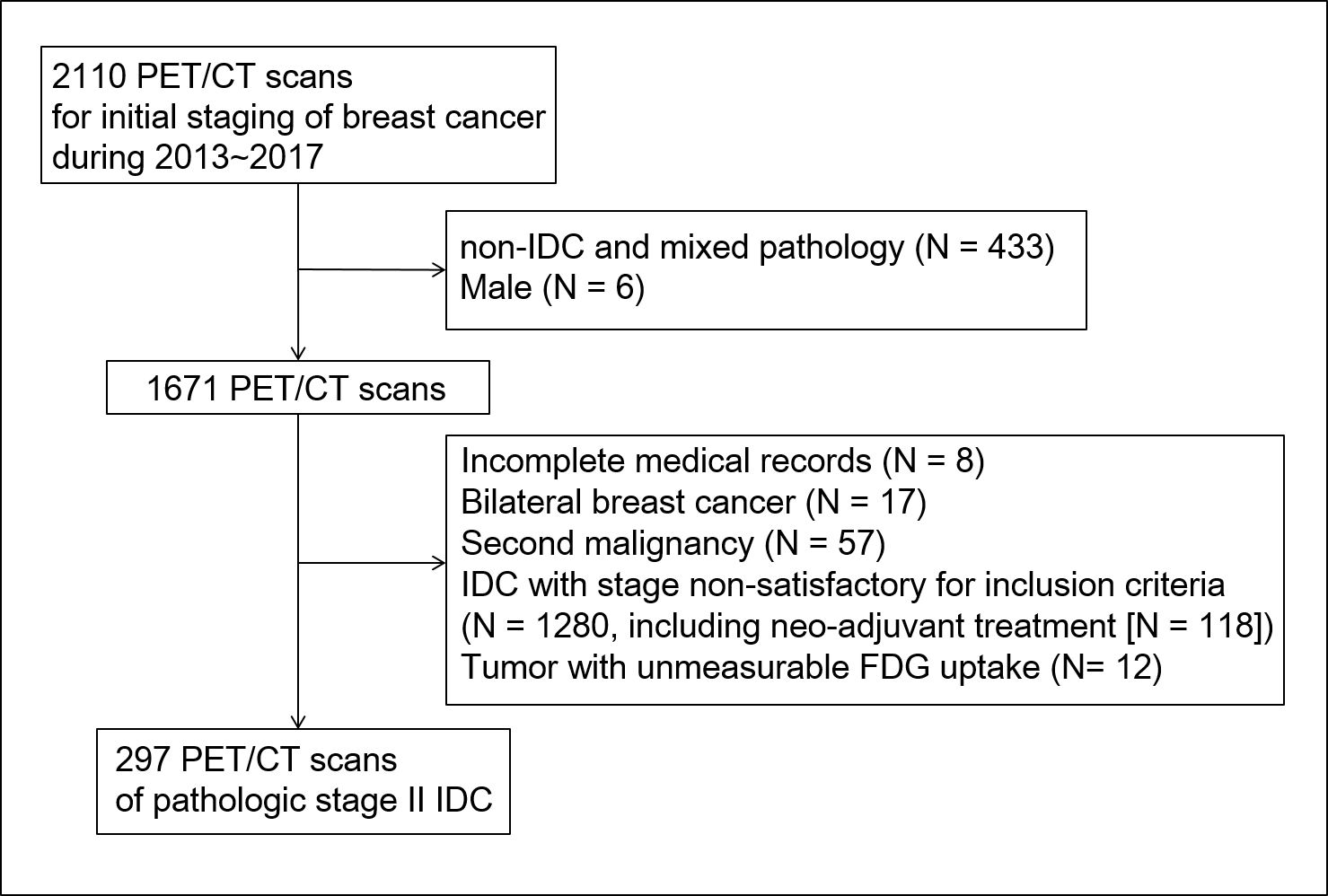

Supplement: Supplementary file 1 — Additional file 1: Supplement 1. Flowchart of study population. [file 40644_2022_519_MOESM1_ESM.tif]
