# Supplementary material for: Prognostic impact of 18F-FDG PET/CT in pathologic stage II invasive ductal carcinoma of the breast: re-illuminating the value of PET/CT in intermediate-risk breast cancer
Source: Cancer Imaging. 2023 Jan 4;23:2. doi: 10.1186/s40644-022-00519-6 (PMC9811771; doi:10.1186/s40644-022-00519-6)
Supplement: Supplementary file 2 — Additional file 2: Supplement 2. Characteristics of recurrence (N = 35). [file 40644_2022_519_MOESM2_ESM.docx]

**Supplement 2. Characteristics of recurrence (N = 35)**

| **Recurrence** | **Location** | **N** | **Confirm methods** |
| --- | --- | --- | --- |
| Locoregional recurrence | breast | 8 | pathology |
|  | Axillary LN | 3 | pathology |
|  | Internal mammary LN | 2 | imaging |
| Distant metastasis | bone | 4 | imaging |
|  | liver | 3 | imaging |
|  | lung | 5 | pathology |
|  |  | 6 | imaging |
|  | pericardium | 1 | pathology |
|  | pleura | 1 | pathology |
|  | skin | 1 | pathology |
| Locoregional recurrence with distant metastasis | Axillary LN and bone | 1 | Pathology for LN and imaging for bone |
